# Emerging Non-Thermal Food Processing Technologies: Editorial Overview

**DOI:** 10.3390/foods11071003

**Published:** 2022-03-29

**Authors:** Asgar Farahnaky, Mahsa Majzoobi, Mohsen Gavahian

**Affiliations:** 1 Biosciences and Food Technology, School of Science, RMIT University, Bundoora West Campus, Plenty Road, Melbourne, VIC 3083, Australia; asgar.farahnaky@rmit.edu.au (A.F.); mahsa.majzoobi@rmit.edu.au (M.M.); 2 Department of Food Science, College of Agriculture, National Pingtung University of Science and Technology, 1, Shuefu Road, Neipu, Pingtung 91201, Taiwan

According to the statistics, there is a strong consumer trend towards high-quality and healthy foods with “fresh-like” characteristics. On the other hand, thermal processing technologies, especially conventional ones, negatively affect the sensory and nutritional properties of foods. At the same time, the limited shelf-life and safety concerns of fresh foods necessitate food processing. Therefore, scientists are exploring the possibility of using nonthermal technologies for various purposes, such as shelf-life extension and the improvement of safety. These technologies include cold plasma, plasma-activated water, pulsed electric fields, moderate electric fields, ultrasound, high-pressure technologies, and other innovative approaches. However, their applicability and scalability are still under intensive investigation.

In this sense, a Special Issue entitled “Emerging Non-Thermal Food Processing Technologies” was launched in *Foods* (MDPI) to provide a forum for researchers to communicate some of their most recent findings on the applications of emerging thermal technologies for food processing. A total number of 17 manuscripts were submitted to this Special Issue from different regions of the world (Figure 1). According to the peer review process, 11 original research articles were included in this Special Issue of *Foods*.

Among the research papers published in this Special Issue, an attractive study investigated the effects of ultra-high-pressure homogenization technology treatment (UHPH) on the structural characteristics of egg yolk granules. The authors observed the high stability of yolk granules during UHPH and reported a restructuration of the granules via the generation of a protein network while the protein profile and proximate composition remained unchanged in a single pass of up to 300 MPa [1]. Another research team conducted an interesting study on the impacts of dual-frequency (28 and 40 kHz) ultrasound-assisted thawing on *Pseudosciaena crocea* (large yellow croaker) to elaborate on the effects of such a process on the microstructure, thawing rate, and quality properties. They reported that dual-frequency sonication can improve the thawing rate without compromising the water-holding capacity, color, texture, and water distribution, while inhibiting the disruption of the microstructure [2]. In another interesting study, the impact of cold plasma on the spores of naturally present fungi as well as the physical and chemical properties of sun-dried tomatoes was investigated. The germination of Aspergillus spores was found to be “species-dependent”. According to the results of this paper, this process inactivated spores by percentages of 88 for *A. rugulovalvus* and 32 for *A. niger*, indicating them to be sensitive and resistant strains, respectively. This research also elaborated on the sporicidal effect of cold plasma on *A. rugulovalvus*. Cold plasma was found to be a promising tool for dried tomato decontamination (in terms of spores) without compromising the physicochemical aspects of the product (e.g., pH and water activity), while increasing the lycopene content significantly [3]. Another paper explained that “pre-crystallization of nougat by seeding with cocoa butter crystals enhances the bloom stability of nougat pralines”. This informative study showed that the precrystallization of nougat via seeding with cocoa butter crystals, under controlled conditions, can enhance the physical storage stability of pralines and delay the onset of fat blooming [4]. A research team explored the “mathematical modelling of ultrasound-assisted extraction kinetics of bioactive compounds from artichoke by-products”. This investigation showed that both sonication and temperature can significantly affect the yield of bioactive compounds during the bioactive extraction process from the stem of artichoke. It also proposed models that can be used for process prediction [5]. Another study explored the application of ultraviolet light-emitting diodes (UV-LEDs) to inactivate *Listeria monocytogenes*, *Escherichia coli*, *Bacillus subtilis*, and *Salmonella enterica* Serovar Typhimurium in powdered food ingredients. This innovative study proposed UV-LEDs as alternatives to UV technology, as they have the potential to enhance the decontamination efficiency [6]. Another group of researchers studied the effects of nitrogen-assisted high-pressure processing on the quality indices and microbial quality of bell peppers (fresh-cut). This food processing approach yielded a product with a greater microbial reduction and better microbiological stability during storage [7]. Furthermore, a manuscript discussed the results of a well-designed study on the impact of centrifugal block freeze crystallization (CBFC) on the quality properties of different types of berry juices. CBFC makes it feasible to obtain greater amounts of solutes, in comparison with the original sample, while intensifying the fresh juices’ natural colors and increasing the phenolic compounds and antioxidant capacity in the final products [8]. Besides, a research team conducted an attractive study on high-hydrostatic-pressure technology. They investigated the antioxidant and anti-inflammatory effects of broccoli treated with high hydrostatic pressure in cell models, and reported that this emerging nonthermal technology can enhance the isothiocyanate content of broccoli with antioxidant anti-inflammatory effects, providing an innovative strategy with which to develop healthy food products in the future [9]. Moreover, an insightful manuscript reported a significant improvement in the antioxidant capacity of puffed turmeric by the application of high hydrostatic pressure extraction. This study elaborated on the effects of processing parameters and reported that 400 MPa for 20 min with 70% ethanol were the optimal extraction conditions for the highest antioxidant activity [10]. In another attractive study, researchers investigated the antibacterial efficacy and mechanisms of plasma-activated water (PAW) against *Salmonella enterica* serovar Enteritidis on eggshells, and reported that PAW is a promising decontamination technology due to the presence of ozone, nitrate, and other reactive species. They also provided images from scanning electron microscopes to elaborate on the effects of this technology on eggshells [11]. 

In summary, the findings published in this Special Issue clearly indicate both the breadth and depth of the recent studies on nonthermal processing technologies. They cover both a wide range of foods products as well as nonthermal technologies. Consumer demand for less-processed foods with a fresh flavor and delicate texture, plus better nutritional quality, are the main driving forces behind the continued research in this area, and it is expected that these technologies will be acquired at a higher pace by the food industry over the next decade.

## Figures and Tables

**Figure 1:**
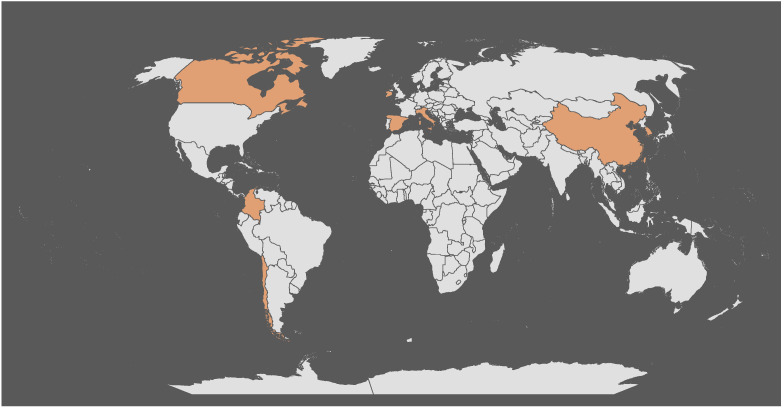
Origins of the submitted papers to this Special Issue based on the affiliations of the authors of the accepted papers. They include Canada, Chile, China, Spain, Taiwan, South Korea, Columbia, Ireland, and Italy.
